# Maternal Cardiometabolic Risk Factors in Pregnancy and Offspring Blood Pressure at Age 2 to 18 Years

**DOI:** 10.1001/jamanetworkopen.2025.9205

**Published:** 2025-05-08

**Authors:** Zhongzheng Niu, Ako Adams Ako, Sarah Dee Geiger, Caitlin G. Howe, Wei Perng, Rachana Singh, Margaret R. Karagas, Amy J. Elliott, Andrea Cassidy-Bushrow, Carlos A. Camargo, Keia Sanderson, Cynthia T. McEvoy, Emily Oken, Dana Dabelea, Tina V. Hartert, Brian Carter, Annemarie Stroustrup, Andrea Lampland, Thomas G. O’Connor, Semsa Gogcu, Mark L. Hudak, Lyndsey E. Shorey-Kendrick, Qi Zhao, Yu Ni, Jeffrey VanWormer, Assiamira Ferrara, Monique Hedderson, Yeyi Zhu, Akram Alshawabkeh, Jose Cordero, Daphne Koinis-Mitchell, Susan Carnell, Carrie V. Breton, Theresa M. Bastain, Shohreh F. Farzan

**Affiliations:** 1 Division of Environmental Health, Department of Population and Public Health Sciences, Keck School of Medicine, University of Southern California, Los Angeles; 2 Department of Epidemiology and Environmental Health, School of Public Health and Health Professions, State University of New York at Buffalo; 3 Department of Pediatrics, Children’s Hospital at Montefiore, Bronx, New York; 4 Department of Kinesiology and Community Health, University of Illinois Urbana-Champaign; 5 Department of Epidemiology, Geisel School of Medicine, Dartmouth College, Lebanon, New Hampshire; 6 Department of Epidemiology, Colorado School of Public Health, University of Colorado Anschutz Medical Campus, Aurora; 7 Department of Pediatrics, Tufts University School of Medicine, Boston, Massachusetts; 8 Avera Research Institute, Sioux Falls, South Dakota; 9 Department of Public Health Sciences, Henry Ford Health, Detroit, Michigan; 10 Department of Emergency Medicine, Massachusetts General Hospital, Harvard Medical School, Boston; 11 Department of Medicine, University of North Carolina at Chapel Hill; 12 Department of Pediatrics, Papé Pediatric Research Institute, Oregon Health & Science University, Portland; 13 Division of Chronic Disease Research Across the Lifecourse, Department of Population Medicine, Harvard Medical School and Harvard Pilgrim Health Care Institute, Boston, Massachusetts; 14 Departments of Medicine and Pediatrics, Vanderbilt University Medical Center, Nashville, Tennessee; 15 Department of Pediatrics-Neonatology, Children’s Mercy Hospital, Kansas City, Missouri; 16 Division of Neonatology, Department of Pediatrics, Cohen Children’s Medical Center at Northwell Health, New York, New York; 17 Department of Neonatology, Children’s Minnesota, St Paul; 18 Department of Psychiatry, University of Rochester Medical Center, Rochester, New York; 19 Division of Neonatology, Department of Pediatrics, Wake Forest University School of Medicine, Winston-Salem, North Carolina; 20 Department of Pediatrics, University of Florida College of Medicine, Gainesville; 21 Department of Preventative Medicine, College of Medicine, University of Tennessee Health Science Center, Memphis; 22 Department of Environmental and Occupational Health Sciences, School of Public Health, University of Washington, Seattle; 23 School of Public Health, San Diego State University, San Diego, California; 24 Marshfield Clinic Research Institute, Marshfield, Wisconsin; 25 Division of Research, Kaiser Permanente Northern California, Pleasanton, California; 26 Department of Civil and Environmental Engineering, Northeastern University, Boston, Massachusetts; 27 Department of Epidemiology and Biostatistics, College of Public Health, University of Georgia, Athens; 28 Departments of Pediatrics and Psychiatry and Human Behavior, Warren Alpert Medical School, Brown University, Providence, Rhode Island; 29 Division of Child and Adolescent Psychiatry, Department of Psychiatry and Behavioral Sciences, School of Medicine, Johns Hopkins University, Baltimore, Maryland; 30 Department of Pediatrics and Human Development, College of Human Medicine, Michigan State University, East Lansing

## Abstract

**Importance:**

Higher blood pressure in early life may signal cardiovascular disease over the life course, but determinants of blood pressure in early life are poorly understood.

**Objective:**

To examine the association of maternal cardiometabolic risk factors during pregnancy with offspring blood pressure from age 2 to 18 years and explore whether the association is modified by offspring sex and race and ethnicity.

**Design, Setting, and Participants:**

This cohort study analyzed data from the Environmental Influences on Child Health Outcomes program between January 1, 1994, and March 31, 2023. Three common maternal cardiometabolic risk factors during pregnancy were examined: prepregnancy obesity, gestational diabetes, and hypertensive disorders of pregnancy (HDP).

**Exposure:**

Maternal cardiometabolic risk factors were retrieved and harmonized from medical records and questionnaires.

**Main Outcomes and Measures:**

Offspring systolic blood pressure (SBP) and diastolic blood pressure (DBP) percentiles adjusted for age, sex, and height were calculated.

**Results:**

Among 12 480 mother-offspring pairs (mean [SD] maternal age during pregnancy, 29.9 [6.4] years; 856 of 12 303 identifying as Asian [7.0%]; 1908 as Black [15.5%]; 2305 as Hispanic [18.7%]; 6522 as White [52.3%], and 712 as other [5.8%] race and ethnicity), at least 1 maternal cardiometabolic risk factor was present in 5537 (44.4%), with prepregnancy obesity being the most prevalent (3072 [24.6%]), followed by HDP (1693 [13.6%]) and gestational diabetes (805 [6.5%]). Offspring born to mothers with any cardiometabolic risk factors had higher SBP (4.88 percentile points; 95% CI, 3.97-5.82 percentile points) and higher DBP (1.90 percentile points; 95% CI, 1.15-2.64 percentile points) at their first blood pressure measurement, after adjusting for potential confounders, compared with their counterparts without any risk factors. Hypertensive disorders of pregnancy, alone or with either prepregnancy obesity or gestational diabetes, was significantly associated with higher offspring blood pressure. These associations were generally more significant among female compared with male offspring and among Black compared with other racial and ethnic groups. Among 6015 offspring who had 2 or more blood pressure measures, maternal cardiometabolic risk factors were associated with an increased rate of blood pressure change from age 2 to 18 years (SBP percentile, 0.5 [95% CI, 0.2-0.8] per year; DBP percentile, 0.7 [95% CI 0.5-1.0] per year).

**Conclusions and Relevance:**

These findings suggest that protecting pregnant individuals from cardiometabolic risk factors may promote healthier blood pressure in the next generation.

## Key Points

**Question:**

What is the association between combinations of maternal cardiometabolic risk factors during pregnancy and offspring blood pressure?

**Findings:**

In this cohort study of 12 480 mother-offspring pairs, maternal cardiometabolic risk factors were significantly associated with a 4.88- and 1.90-percentile point higher systolic blood pressure and diastolic blood pressure in their offspring, respectively. A combination of hypertensive disorders of pregnancy with prepregnancy obesity or gestational diabetes mellitus was significantly associated with higher blood pressure and higher rates of blood pressure longitudinal change from age 2 to 18 years.

**Meaning:**

These findings suggest that protecting pregnant individuals from cardiometabolic risk factors may promote healthier blood pressure in the next generation.

## Teaser

This cohort study examines the association of maternal cardiometabolic risk factors during pregnancy with offspring blood pressure from age 2 to 18 years and whether offspring sex or race and ethnicity modifies the association.

## Introduction

High blood pressure in childhood has been associated with an increased risk of hypertension and other cardiovascular diseases in later life.^1,2,3^ Preventing high blood pressure in childhood, therefore, is a promising target to improve cardiovascular health across the life course. However, risk factors for high blood pressure in early life remain poorly understood, and growing evidence has associated them with an adverse intrauterine environment.^4,5,6,7^

The intrauterine environment is influenced by the mother’s health before and during pregnancy. Maternal cardiometabolic risk factors, particularly prepregnancy obesity, gestational diabetes, and hypertensive disorders of pregnancy (HDP), are among the most common complications that may contribute to an adverse environment for the fetus. Their prevalence has been increasing among the US pregnant population. In 2019, these 3 maternal cardiometabolic risk factors were estimated to be present in more than 50% of the US pregnant population.^8^ Previous studies have found that higher maternal prepregnancy body mass index (BMI), gestational diabetes, and HDP are each individually associated with higher offspring blood pressure.^9,10,11,12^ Although largely examined in isolation, these risk factors tend to aggregate and may together create an adverse intrauterine environment.^13^ However, to what extent various combinations and the accumulation of individual maternal cardiometabolic risk factors are associated with childhood blood pressure remains poorly understood. Additionally, most previous studies focused on blood pressure measures in late childhood or adolescence (aged 8-17 years), leaving a crucial gap in understanding the developmental trajectory of blood pressure from early childhood (aged <8 years).

Using data from the Environmental Influences on Child Health Outcomes (ECHO) program, we aimed to examine the association of the 3 most common maternal cardiometabolic risk factors with children’s blood pressure, particularly early measures, to fill the research gap of blood pressure in early childhood. We tested the hypothesis that combinations of maternal cardiometabolic risk factors may be additively associated with increased offspring blood pressure. We further examined their associations with longitudinal blood pressure changes from age 2 to 18 years. Prior research has shown that the prevalence of childhood hypertension and maternal cardiometabolic conditions varies by sex and race and ethnicity, suggesting that the same intrauterine exposure may not uniformly influence offspring blood pressure across all groups.^14,15^ Therefore, we further investigated whether offspring sex and race and ethnicity would modify the association of maternal cardiometabolic risk factors with offspring blood pressure. It is important to note that while sex differences in blood pressure may reflect biological variations, disparities by race and ethnicity, which are socially constructed categories, reflect underlying social determinants of health.^16,17^

## Methods

### Population

This cohort study examined data from ECHO, a collaborative research program with more than 50 000 children and families from 69 birth and pediatric cohorts from 1994 to 2023 across the US.^18,19^ All participants provided written informed consent in accordance with protocols approved by institutional review boards of individual cohorts and the ECHO program.^18^ This study followed the Strengthening the Reporting of Observational Studies in Epidemiology (STROBE) reporting guideline.

The ECHO cohorts use common protocols to assess a broad range of metrics of child health, including cardiometabolic health of both the children and their mothers during pregnancy. Details on study design of the ECHO program and each specific cohort (eg, inclusion criteria) have been reported in previous publications and can be found on the ECHO website.^18,19^ For this analysis, the inclusion criteria were based on data availability of (1) the child having at least 1 blood pressure measure at any age from 2 to 18 years and (2) the mother having a record indicating the presence or absence of at least 1 of the maternal cardiometabolic risk factors. For longitudinal analysis of blood pressure changes over time, only children with 2 or more measures at different years of age were included. We also compared the characteristics between individuals included and excluded in the longitudinal study. A detailed flow diagram of the sample is provided in eFigure 1 in Supplement 1.

### Maternal Cardiometabolic Risk Factors During Pregnancy

Information on maternal cardiometabolic risk factors was retrieved from cohort-specific sources, including medical record review, self-report from questionnaires, and physical measurements at study visits. The ECHO centralized data core reviewed and harmonized data across cohorts into the final variables.^19^ Prepregnancy obesity was defined as a BMI of 30 or higher using body weight (in kilograms) divided by height (in meters squared) measured or self-reported between 12 months before conception and the first trimester (13 gestational weeks). Gestational diabetes mellitus was defined as new-onset diabetes diagnosed during pregnancy. Hypertensive disorders of pregnancy included gestational hypertension (ie, systolic blood pressure [SBP] ≥140 mm Hg or diastolic blood pressure [DBP] ≥90 mm Hg on 2 occasions at least 4 hours apart after 20 gestational weeks) and preeclampsia (ie, hypertension with the presence of organ damage, such as proteinuria). Cases of essential hypertension and type 1 or 2 diabetes were excluded from the analyses to focus on emergent maternal cardiometabolic complications during pregnancy. We defined any maternal cardiometabolic risk factors as the presence of at least 1 of prepregnancy obesity, gestational diabetes, or HDP. To fully examine the effect of different risk factors combinations, we constructed 8 nominal categories based on possible combinations of yes and no for each of prepregnancy obesity, gestational diabetes, and HDP (no risk factors, obesity only, HDP only, gestational diabetes only, obesity plus HDP, obesity plus gestational diabetes, HDP plus gestational diabetes, and all 3 risk factors).

### Offspring Blood Pressure

Offspring blood pressure was measured during study visits or extracted from medical records for each cohort. The ECHO centralized data core reviewed and harmonized age-, sex-, and height-specific blood pressure percentiles according to American Academy of Pediatrics guidelines for screening and management of high blood pressure in children and adolescents.^19,20^ When there was more than 1 blood pressure measurement at the same age, we used the mean as the blood pressure measured at that age to improve precision. When blood pressure measures were available at different years of age, we used the blood pressure measure at the youngest age (as a single time point outcome) to focus our analysis on younger children. We used blood pressure measures at all ages for longitudinal analysis in mixed-effects models (details provided in the Statistical Analysis).

### Covariates

The following covariates were considered as potential confounders based on directed acyclic graphs^21^ (eFigure 2 in Supplement 1): maternal age at the index pregnancy, self-identified race and ethnicity (Hispanic, non-Hispanic Asian, non-Hispanic Black, non-Hispanic White [hereafter referred to as Asian, Black, and White, respectively], or other [Alaska Native, American Indian, Native Hawaiian, Pacific Islander, unspecified, or multiracial]), education, household income, marital status, prenatal smoking status, and parity. Education and income, collected from questionnaires in each cohort with potentially differential levels, were harmonized by the centralized data core. Missing covariates were iteratively imputed using the package mice in R, version 4.0.1 (R Project for Statistical Computing) to preserve power while reducing bias vs simpler methods (eg, mean imputation).^22^ Offspring body weight was not considered as a confounder given its potential mediating role on the association of maternal risk factors with offspring blood pressure.

### Statistical Analysis

We used means and standard deviations to describe SBP/DBP and other continuous variables and counts and percentages to describe the prevalence of maternal cardiometabolic risk factors and combinations and other categorical variables. We used linear regression to analyze the association of maternal cardiometabolic risk factors with offspring blood pressure at the youngest age in separate models in which independent variables were (1) any or none of the 3 maternal risk factors, (2) the various risk factor combinations (8 categories), and (3) each of the 3 risk factors per se. To analyze potential effect modification by offspring sex and race and ethnicity, we included product terms of maternal cardiometabolic risk factors with each effect modifier. We also stratified the analysis by levels of modifiers. To analyze the longitudinal changes in blood pressure from age 2 to 18 years, we used mixed-effects models that included a random intercept and a random slope of children’s age, adjusting for the same sets of confounders. An unstructured covariance matrix was used to maximize flexibility in random-effects modeling and to minimize misspecification. An interaction term between age and risk factors was included. The coefficient of the interaction term quantified the degree to which maternal factors modified the rate of blood pressure change over time. A positive coefficient suggested that as children grew, the influence of maternal factors on the rate of blood pressure change became more pronounced. To assess cohort effects, we conducted leave-one-out analyses in which all models were repeated while omitting 1 cohort from the analysis at a time. In addition, we used a mixed-effects model as a sensitivity analysis to include cohort as a cluster factor. The significance level was set at a 2-sided *P* < .05 (ie, the *P* value was not adjusted for multiple comparison). All analyses were conducted using R, version 4.0.1.

## Results

The overall study population included 12 480 mothers (mean [SD] age, 29.9 [6.41] years; 856 of 12 303 identifying as Asian [7.0%]; 1908 as Black [15.5%]; 2305 as Hispanic [18.7%]; 6522 as White [52.3%], and 712 as other [5.8%] race and ethnicity) (Table 1). A total of 5537 mothers (44.4%) had at least 1 maternal cardiometabolic risk factor. Prepregnancy obesity was the most prevalent risk factor (3072 mothers [24.6%]), followed by HDP (1693 mothers [13.6%]) and gestational diabetes (805 mothers [6.5%]). Most offspring (6083 female [48.7%] and 6397 male [51.3%]; 573 identified as Asian [4.7%], 1826 as Black [14.9%], 2747 as Hispanic [22.4%], 5921 as White [48.3%], and 1194 as other [9.7%] race and ethnicity) had their first blood pressure measure between age 2 and 5 years (8815 children [70.6%]). Offspring born to mothers with any risk factors had higher sex-, age-, and height-adjusted SBP percentiles (mean [SD], 61.4 [26.0]) and DBP percentiles (mean [SD], 70.5 [20.5]) than their counterparts born to mothers without any risk factors (mean [SD]: SBP, 56.7 [26.4]; DBP, 68.7 [20.8]). Detailed descriptive statistics for blood pressure percentiles by each risk factor and their combination and demographic factors are provided in eTable 1 in Supplement 1. Blood pressure distributions by cohort are shown in eFigure 3 in Supplement 1.

**Table 1.** Characteristics in the Overall Population and by the Presence of Maternal Cardiometabolic Risk Factors During Pregnancy

Characteristic	Individuals, No. (%)			*P* value^b^
	Overall (N = 12 480)	No maternal risk factors (n = 6943)^a^	Any maternal risk factors (n = 5537)^a^	
**Maternal demographics**				
Age at pregnancy, mean (SD), y	29.9 (6.4)	30.1 (6.3)	29.8 (6.6)	.003
Race and ethnicity^c^				
Hispanic	2305 (18.7)	1170 (17.1)	1135 (20.8)	<.001
Non-Hispanic Asian	856 (7.0)	580 (8.5)	276 (5.1)	
Non-Hispanic Black	1908 (15.5)	904 (13.2)	1004 (18.4)	
Non-Hispanic White	6522 (53.0)	3836 (56.1)	2686 (49.2)	
Other^d^	712 (5.8)	353 (5.2)	359 (6.6)	
Missing	177	100	77	
Education				
High school or lower	2425 (20.8)	1184 (17.8)	1241 (24.6)	<.001
Some college, no degree	2971 (25.5)	1486 (22.4)	1485 (29.5)	
College degree	3781 (32.4)	2332 (35.1)	1449 (28.8)	
Master’s degree or higher	2486 (21.3)	1634 (24.6)	852 (16.9)	
Missing	817	307	510	
Family annual income, $				
<30 000	1702 (36.2)	817 (32.2)	885 (41)	<.001
30 000-49 999	536 (11.4)	251 (9.9)	285 (13)	
50 000-74 999	525 (11.2)	275 (10.8)	250 (12)	
75 000-99 999	307 (6.5)	176 (6.9)	131 (6.0)	
≥100 000	1183 (25.1)	770 (30.4)	413 (19)	
Do not know	448 (9.5)	246 (9.7)	202 (9.3)	
Missing	7779	4408	3371	
Marital status				
Married	6599 (79.5)	4107 (82.5)	2492 (75.2)	<.001
Unmarried	1695 (20.4)	871 (17.5)	824 (24.8)	
Missing	4186	1965	2221	
Parity				
Nulliparous	2782 (24.9)	1736 (27.4)	1046 (21.6)	<.001
Parous	8395 (75.1)	4592 (72.6)	3803 (78.4)	
Missing	1303	615	688	
Smoking during pregnancy				
No	10 817 (91.2)	6094 (92.4)	4723 (89.7)	<.001
Yes	1044 (8.8)	504 (7.6)	540 (10.3)	
Missing	619	345	274	
Prepregnancy obesity				
No	8584 (73.6)	6574 (100)	2010 (39.6)	<.001
Yes	3072 (26.4)	0	3072 (60.4)	
Missing	824	369	455	
Gestational diabetes				
No	10 226 (92.7)	6943 (100)	3283 (80.3)	<.001
Yes	805 (7.3)	0	805 (19.7)	
Missing	1449	0	1449	
HDP				
No	10 421 (86.0)	6943 (100)	3478 (67.3)	<.001
Yes	1693 (14.0)	0	1693 (32.7)	
Missing	366	0	366	
**Offspring characteristics**				
Sex				
Female	6083 (48.7)	3402 (49.0)	2681 (48.4)	.53
Male	6397 (51.3)	3541 (51.0)	2856 (51.6)	
Race and ethnicity				
Hispanic	2747 (22.4)	1443 (21.2)	1304 (24.0)	<.001
Non-Hispanic Asian	573 (4.7)	409 (6.0)	164 (3.0)	
Non-Hispanic Black	1826 (14.9)	860 (12.6)	966 (17.8)	
Non-Hispanic White	5921 (48.3)	3488 (51.2)	2433 (44.7)	
Other^d^	1184 (9.7)	613 (9.0)	571 (10.5)	
Missing	229	130	99	
Age category at first blood pressure measure, y				
2-5	8815 (70.6)	5196 (74.8)	3619 (65.4)	<.001
6-10	2291 (18.3)	1016 (14.6)	1275 (23.0)	
11-18	1374 (11.0)	731 (10.5)	643 (11.6)	
Blood pressure percentile, mean (SD)				
SBP	58.8 (26.4)	56.7 (26.4)	61.4 (26.0)	<.001
DBP	69.5 (20.7)	68.7 (20.8)	70.5 (20.5)	<.001

Abbreviations: DBP, diastolic blood pressure; HDP, hypertensive disorders of pregnancy; SBP, systolic blood pressure.

^a^ Maternal risk factors refer to prepregnancy obesity, gestational diabetes, and HDP.

^b^ The *t* test and *F* test were used to compare continuous variables, and χ^2^ test was used to compare categorical variables.

^c^ Race and ethnicity were considered to reflect social determinants of health that may have implications for maternal complications and offspring blood pressure.

^d^ Other included Alaska Native, American Indian, Native Hawaiian, Pacific Islander, unspecified, or multiracial. These categories were combined due to small numbers.

After adjusting for potential confounders, differences in offspring blood pressure percentiles by maternal cardiometabolic risk factors remained statistically significant (Table 2). Compared with offspring born to mothers without any cardiometabolic risk factors, those born to mothers with any risk factors had higher SBP (4.88 percentile points; 95% CI, 3.97-5.82 percentile points) and higher DBP (1.90 percentile points; 95% CI, 1.15-2.64 percentile points). In examining various combinations of cardiometabolic risk factors, HDP alone or in combination with either prepregnancy obesity or gestational diabetes was significantly associated with higher offspring blood pressure. For SBP, the largest difference was among mothers with both HDP and prepregnancy obesity (adjusted β, 7.31; 95% CI, 4.99-9.62), followed by the combination of HDP and gestational diabetes (adjusted β, 6.19; 95% CI, 0.24-12.14). For DBP, the largest difference was among mothers with a combination of HDP and gestational diabetes (adjusted β, 5.03; 95% CI, 0.30-9.75), followed by a combination of HDP and prepregnancy obesity (adjusted β, 3.61; 95% CI, 1.77-5.46). Gestational diabetes mellitus alone was not associated with DBP (adjusted β, 0.00; 95% CI, −2.12 to 2.10). The presence of all 3 cardiometabolic risk factors (116 participants [0.9%]) was associated with a higher SBP (adjusted β, 3.41; 95% CI, −1.37 to 8.19) and DBP (adjusted β, 2.98; 95% CI, −0.82 to 6.79), although the difference was not statistically significant.

**Table 2.** Association Between Maternal Cardiometabolic Risk Factors During Pregnancy and Offspring Sex-, Age-, and Height-Adjusted Blood Pressure Percentile (N = 12 480)

Maternal cardiometabolic risk factor	Mothers, No. (%)	SBP, β (95% CI)^a^		DBP, β (95% CI)^a^	
		Unadjusted	Adjusted	Unadjusted	Adjusted
Any factor					
None	6943 (55.6)	0 [Reference]	0 [Reference]	0 [Reference]	0 [Reference]
Any	5537 (44.4)	4.71 (3.78 to 5.63)	4.88 (3.97 to 5.82)	1.79 (1.06 to 2.52)	1.90 (1.15 to 2.64)
Combinations					
None	6943 (55.6)	0 [Reference]	0 [Reference]	0 [Reference]	0 [Reference]
Obesity only^b^	1871 (15.0)	3.21 (1.87 to 4.55)	3.18 (1.83 to 4.52)	2.15 (1.09 to 3.20)	1.75 (0.68 to 2.83)
HDP only	874 (7.0)	2.90 (1.06 to 4.75)	3.42 (1.59 to 5.24)	1.57 (0.11 to 3.02)	1.84 (0.38 to 3.31)
Gestational diabetes only	396 (3.2)	3.68 (1.03 to 6.34)	2.58 (−0.08 to 5.24)	0.74 (−1.35 to 2.83)	0.00 (−2.12 to 2.10)
Obesity + HDP^b^	525 (4.2)	7.38 (5.05 to 9.71)	7.31 (4.99 to 9.62)	4.04 (2.20 to 5.88)	3.61 (1.77 to 5.46)
Obesity + gestational diabetes^b^	219 (1.8)	6.30 (2.77 to 9.83)	5.72 (2.21 to 9.23)	3.25 (0.45 to 6.04)	2.29 (−0.53 5.46)
HDP + gestational diabetes	74 (0.6)	6.74 (0.73 to 12.75)	6.19 (0.24 to 12.14)	5.62 (0.89 to 10.35)	5.03 (0.30 to 9.75)
All 3	116 (0.9)	4.38 (−0.44 to 9.19)	3.41 (−1.37 to 8.19)	3.45 (−0.34 to 7.24)	2.98 (−0.82 to 6.79)
Gestational diabetes^c^					
No	10 226 (81.9)	0 [Reference]	0 [Reference]	0 [Reference]	0 [Reference]
Yes	805 (6.5)	3.56 (1.68 to 5.45)	2.63 (0.74 to 4.53)	1.53 (0.04 to 3.02)	0.85 (−0.66 to 2.37)
HDP^c^					
No	10 421 (83.5)	0 [Reference]	0 [Reference]	0 [Reference]	0 [Reference]
Yes	1693 (13.6)	3.37 (2.01 to 4.72)	3.59 (2.25 to 4.93)	2.26 (1.20 to 3.32)	2.28 (1.21 to 3.35)
Obesity^b^^,^^c^					
No	8584 (68.8)	0 [Reference]	0 [Reference]	0 [Reference]	0 [Reference]
Yes	3072 (31.2)	4.02 (2.94 to 5.10)	3.97 (2.87 to 5.07)	2.37 (1.52 to 3.22)	2.04 (1.17 to 2.92)

Abbreviations: DBP, diastolic blood pressure; HDP, hypertensive disorders of pregnancy; SBP, systolic blood pressure.

^a^ The results are for 1-unit change in blood pressure percentile from different models with different definitions of maternal cardiometabolic risk factors or combinations that have their own reference group. The adjusted model included maternal age, race and ethnicity, education, income, marital status, parity, and smoking during pregnancy.

^b^ Obesity refers to prepregnancy obesity.

^c^ These results were derived from separate models (ie, factors not mutually adjusted).

**Figure 1.**
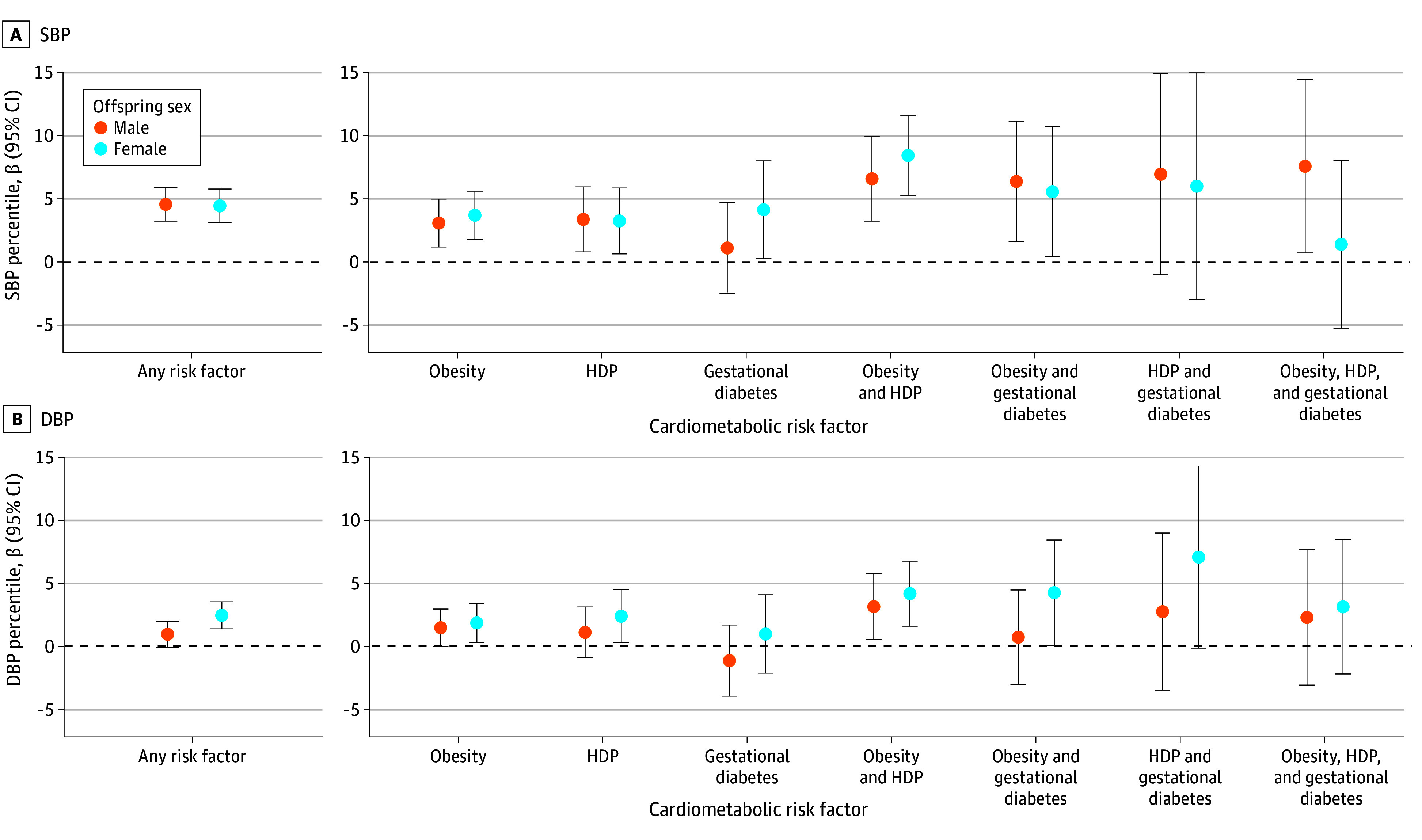
Linear Model With Effect Modification by Offspring Sex The model was adjusted for maternal age, race and ethnicity, education, income, marital status, parity, and smoking during pregnancy. DBP indicates diastolic blood pressure; HDP, hypertensive disorders of pregnancy; obesity, prepregnancy obesity; SBP, systolic blood pressure.

Offspring sex modified the association between cardiometabolic risk factors and offspring DBP (Figure 1; eTable 2 in Supplement 1). The association of any cardiometabolic risk factors with DBP was more pronounced in female offspring (adjusted β, 2.73; 95% CI, 1.64-3.82) than in male offspring (adjusted β, 1.03; 95% CI, −0.08 to 2.13) (*P* for interaction = .03). Although the point estimates for gestational diabetes and HDP were numerically higher in female offspring, neither interaction reached statistical significance. The associations with SBP were comparable between male and female offspring.

**Figure 2.**
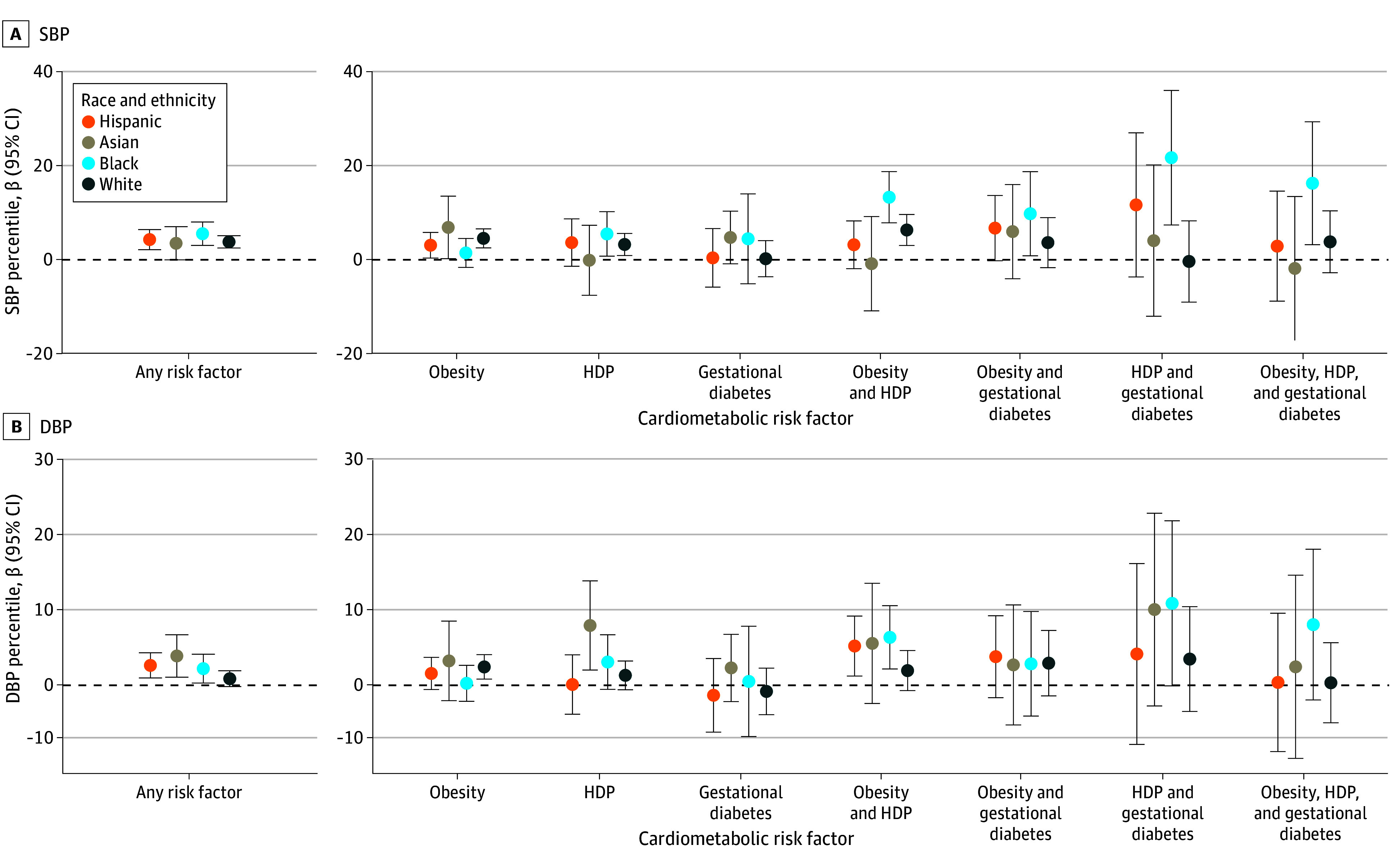
Linear Model With Effect Modification by Race and Ethnicity The model was adjusted for maternal age, education, income, marital status, parity, and smoking during pregnancy. DBP indicates diastolic blood pressure; HDP, hypertensive disorders of pregnancy; obesity, prepregnancy obesity; SBP, systolic blood pressure.

Race and ethnicity modified the association between maternal cardiometabolic risk factors and offspring blood pressure. For each risk factor separately, associations were more pronounced among Black offspring for SBP with gestational diabetes (adjusted β, 7.60 compared with 0.63 among White offspring; *P* for interaction = .01) and HDP (adjusted β, 7.48 compared with 3.42 for White offspring; *P* for interaction = .01) (eTable 3 in Supplement 1). By contrast, the association of prepregnancy obesity with SBP was greater among White offspring (adjusted β, 4.83; 95% CI, 3.19-6.47), followed by Black offspring (adjusted β, 3.66; 95% CI, 0.95-6.38), Hispanic offspring (adjusted β, 3.03; 95% CI, 0.65-5.40), and Asian offspring (adjusted β, 2.88; 95% CI, −2.03 to 7.79) (*P* for interaction = .03). For all combinations of cardiometabolic risk factors, SBP percentiles were highest among Black offspring, except for prepregnancy obesity alone, which was highest among Asian offspring (Figure 2). For DBP percentiles, any cardiometabolic risk factors, prepregnancy obesity, and HDP was highest among Asian offspring, while gestational diabetes alone, HDP combined with either prepregnancy obesity or gestational diabetes, or all 3 combined were highest among Black offspring.

Mothers in the longitudinal analyses were more likely to be Asian, had some college or higher education, and were married, while children were more likely to have a lower SBP percentile and higher DBP percentile at first blood pressure measure compared with those not included in the longitudinal analyses (eTable 4 in Supplement 1). Whereas direct blood pressure measures (in mm Hg) increased as children grew, the sex-, age-, and height-adjusted blood pressure percentiles of our cohort decreased over time. The SBP percentile decreased by 0.5 per year (95% CI, 0.2-0.8 per year) and DBP by 3.4 per year (95% CI, 3.1-3.6 per year). However, among children whose mothers had any cardiometabolic risk factors, the decline in blood pressure percentile over time was less steep for SBP (β for the interaction term, 0.46; 95% CI, 0.15-0.76) and DBP (β for the interaction term, 0.74; 95% CI, 0.46-1.01), thus resulting in higher blood pressure percentiles at older ages compared with offspring of mothers without risk factors (Table 3). For combinations of cardiometabolic risk factors, HDP alone exhibited a significant interaction with offspring age (β, 1.16; 95% CI, 0.66-1.66). Most other combinations had a nonsignificant interaction with age on SBP and DBP.

**Table 3.** Longitudinal Changes of Sex-, Age-, and Height-Adjusted Blood Pressure Percentile by Maternal Cardiometabolic Risk Factors, Interaction Associations With Offspring Age (n = 6015)^a^

Maternal cardiometabolic risk factor	SBP percentile		DBP percentile	
	β (95% CI)	*P* value	β (95% CI)	*P* value
Any factors (vs none)				
Any factors × age	0.46 (0.15 to 0.76)	.01	0.74 (0.46 to 1.01)	<.001
Combinations (vs none)				
Obesity only × age^b^	0.39 (−0.02 to 0.81)	.06	0.14 (−0.23 to 0.52)	.45
Gestational diabetes only × age	0.30 (−0.62 to 1.22)	.53	0.32 (−0.51 to 1.14)	.45
HDP only × age	0.23 (−0.32 to 0.78)	.42	1.16 (0.66 to 1.66)	<.001
(Obesity + HDP) × age^b^	0.45 (−0.35 to 1.26)	.28	0.17 (−0.55 to 0.90)	.64
(Obesity + gestational diabetes) × age^b^	−0.89 (−2.07 to 0.28)	.14	−1.29 (−2.34 to −0.23)	.02
(HDP + gestational diabetes) × age	0.74 (−1.43 to 2.92)	.50	0.91 (−1.02 to 2.84)	.36
All 3 × age	0.64 (−0.99 to 2.27)	.44	0.59 (−0.87 to 2.04)	.43
Obesity (vs nonobesity)^b^				
Obesity × age	0.34 (−0.01 to 0.70)	.06	−0.04 (−0.37 to 0.30)	.83
Gestational diabetes (vs no gestational diabetes)				
Gestational diabetes × age	−0.06 (−0.70 to 0.59)	.86	−0.17 (−0.75 to 0.40)	.56
HDP (vs no HDP)				
HDP × age	0.16 (−0.28 to 0.60)	.47	0.73 (0.34 to 1.13)	<.001

Abbreviations: DBP, diastolic blood pressure; HDP, hypertensive disorders of pregnancy; SBP, systolic blood pressure.

^a^ These results were from different models with different definitions of maternal cardiometabolic risk factors or combinations that each have their own reference group. Models were adjusted for maternal age, race and ethnicity, education, income, marital status, parity, and smoking during pregnancy.

^b^ Obesity refers to prepregnancy obesity.

In sensitivity analysis omitting 1 cohort at a time, the findings were generally robust (eFigure 4 in Supplement 1). The results were also robust when we included cohort as a cluster factor in a random-effects model (eTable 5 in Supplement 1).

## Discussion

In this cohort study, we analyzed different combinations of maternal cardiometabolic risk factors and found that the co-occurrence of HDP with either gestational diabetes or prepregnancy obesity had a profound association with offspring blood pressure compared with each risk factor alone. Among the 3 risk factors, HDP has been the most extensively studied with regard to long-term effects on offspring health,^11,12,23^ including higher blood pressure, lower vascular distensibility, increased vascular stiffness, and retinal arteriolar narrowing.^24,25,26^ Importantly, previous studies found that the associations between maternal cardiometabolic risk factors and high offspring blood pressure were independent of familial and individual adiposity,^27,28,29,30,31^ thus collectively suggesting a possible direct programming effect of intrauterine exposure to HDP and/or gestational diabetes on offspring blood pressure.^26,28,32^ If such a programming effect is causal, achieving a healthier cardiometabolic profile in the childbearing population would be essential to improve cardiovascular health in future generations. The more pronounced DBP associations in female offspring warrant further investigation, given that previous studies on sex differences have been inconsistent.^33,34^

Our longitudinal analysis findings of an increased rate of blood pressure change over time by exposure to maternal cardiometabolic risk factors provides novel evidence. Previous studies found that gestational diabetes is only associated with higher SBP among offspring by age 10 years but not at earlier ages (3 and 7 years).^33^ In a retrospective study, gestational diabetes was also found to be associated with offspring risk of cardiovascular disease by age 40 years.^35^ Collectively, offspring born to mothers with cardiometabolic risk factors not only had a higher blood pressure at a younger age but such differences also were further amplified as they grew up.

Mechanisms underlying the association between maternal cardiometabolic risk factors and offspring blood pressure remain unclear, particularly for the combinations of different risk factors. Most previous studies treated prepregnancy body weight, such as BMI and obesity, as a confounder because of its plausible effect on both gestational diabetes and HDP and higher offspring blood pressure.^29,30^ Our findings that the combinations of prepregnancy obesity and HDP or gestational diabetes are more significantly associated with offspring blood pressure than either alone suggest a potential additive association of prepregnancy obesity with HDP and gestational diabetes. Aside from the possible contributions of shared genetic background and lifestyle, obesity can increase blood pressure, eg, by directly disrupting placental-fetal circulation via sphingolipids, such as ceramides,^36^ or interacting with HDP by inducing angiotensin II–elicited hypertensive responses.^23^ Obesity co-occurring with gestational diabetes may increase insulin resistance and adiponectin suppression, which may subsequently modify fetal vascular tone by diminishing the production of nitric oxide and inducing endothelial dysfunction.^37^

Our findings have important public health implications. In the face of declining generational cardiovascular health in the overall US population,^38^ our results suggest that crucial next steps should include escalating interventions to prevent obesity and enhanced screening and treatment of gestational diabetes and HDP in childbearing populations. Such a move might not only protect mothers from long-term cardiovascular disease risk themselves but also shift the population distribution toward healthier end points in the next generations. Because we have identified an amplified association with offspring blood pressure changes over time by maternal cardiometabolic risk factors and clear evidence on the sequelae of higher childhood blood pressure on cardiovascular disease risk later in life,^1^ targeted screening should be prioritized for young children born to mothers with obesity, gestational diabetes, or HDP. Early intervention for controlling blood pressure, such as lifestyle modification, also may be warranted. The prevalence of maternal cardiometabolic risk factors was higher among Black pregnant individuals. Our findings of a significant association of offspring blood pressure among youths identified as Black further suggest that the racial and ethnic disparities in cardiovascular disease may also have early-life origins. Future studies should consider incorporating social determinants of health, such as the Child Opportunity Index, to further elucidate the interplay of structural factors and maternal-child health.

### Strengths and Limitations

Our study was strengthened by a large, diverse population that enabled us to examine various combinations of maternal cardiometabolic risk factors. Our longitudinal analyses on blood pressure changes from age 2 to 18 years may help to bridge the research gap in understanding long-term associations of an adverse intrauterine environment with blood pressure in childhood.

Our study also has some limitations. First, we treated HDP as an umbrella hypertensive complication, whereas HDP subtypes may each have subtle differences in their association with offspring blood pressure. In addition, BMI is an imperfect measure of adiposity. Second, we lacked information on gestational weight gain, diet, and physical activity, which are also important but were not studied due to the lack of harmonized data. These and other unadjusted factors may induce residual biases. Third, we did not adjust for multiple comparisons because our analyses were hypothesis driven, so there may be a slight increase in type I error.

## Conclusions

In this cohort study of 12 480 mother-offspring pairs, we found that prepregnancy obesity, gestational diabetes, and HDP, alone or in various combinations, were prospectively associated with higher offspring blood pressure at an early age and with an increased rate of blood pressure change from age 2 to 18 years, with the most profound associations with DBP among female offspring and with SBP among Black offspring. Implementing clinical screening and treatment guidelines to identify and treat cardiometabolic risk factors in pregnant individuals may foster better cardiovascular health in the next generation. Clinical screening and treatment guidelines that provide early identification of and protect against cardiometabolic risk factors in the childbearing population may promote healthier blood pressure in the next generation.

## Notes

Supplement 1. **eTable 1.** Offspring Blood Pressure by Demographics and Maternal Cardiometabolic Risk Factors During Pregnancy **eTable 2.** Sex Modification on the Association Between Maternal Cardiometabolic Risk Factors During Pregnancy and Offspring Blood Pressure **eTable 3.** Race and Ethnicity Modification on the association Between Maternal Cardiometabolic Risk Factors During Pregnancy and Offspring Blood Pressure **eTable 4.** Population Characteristics According to Exclusion and Inclusion in Longitudinal Analyses **eTable 5.** Sensitivity Analysis Results Compared With the Original Results **eFigure 1.** Flow Diagram of Population Included in the Analysis. **eFigure 2.** Causal Diagram **eFigure 3.** Blood Pressure Distribution by Cohort **eFigure 4.** Beta and 95% CI From Leave-One-Out Analysis

Supplement 2.
Nonauthor Collaborators

Supplement 3.
Data Sharing Statement
