# Supplementary material for: Maternal Cardiometabolic Risk Factors in Pregnancy and Offspring Blood Pressure at Age 2 to 18 Years
Source: JAMA Netw Open. 2025 May 8;8(5):e259205. doi: 10.1001/jamanetworkopen.2025.9205 (PMC12062903; doi:10.1001/jamanetworkopen.2025.9205)
Supplement: Supplement 1. — eTable 1. Offspring Blood Pressure by Demographics and Maternal Cardiometabolic Risk Factors During Pregnancy eTable 2. Sex Modification on the Association Between Maternal Cardiometabolic Risk Factors During Pregnancy and Offspring Blood Pressure eTable 3. Race and Ethnicity Modification on the association Between Maternal Cardiometabolic Risk Factors During Pregnancy and Offspring Blood Pressure eTable 4. Population Characteristics According to Exclusion and Inclusion in Longitudinal Analyses eTable 5. Sensitivity Analysis Results Compared With the Original Results eFigure 1. Flow Diagram of Population Included in the Analysis. eFigure 2. Causal Diagram eFigure 3. Blood Pressure Distribution by Cohort eFigure 4. Beta and 95% CI From Leave-One-Out Analysis [file jamanetwopen-e259205-s001.pdf]

Supplemental Online Content

Niu Z, Ako AA. Geiger SD, et al. Maternal cardiometabolic risk factors in pregnancy and offspring blood pressure at age 2 to 18 years. *JAMA Netw Open*. 2025;8(5):e259205. doi:10.1001/jamanetworkopen.2025.9205

eTable 1. Offspring Blood Pressure by Demographics and Maternal Cardiometabolic Risk Factors During Pregnancy

eTable 2. Sex Modification on the Association Between Maternal Cardiometabolic Risk Factors During Pregnancy and Offspring Blood Pressure

eTable 3. Race and Ethnicity Modification on the association Between Maternal Cardiometabolic Risk Factors During Pregnancy and Offspring Blood Pressure

eTable 4. Population Characteristics According to Exclusion and Inclusion in Longitudinal Analyses

eTable 5. Sensitivity Analysis Results Compared With the Original Results

eFigure 1. Flow Diagram of Population Included in the Analysis

eFigure 2. Causal Diagram

eFigure 3. Blood Pressure Distribution by Cohort

eFigure 4. Beta and 95% CI From Leave-One-Out Analysis

This supplemental material has been provided by the authors to give readers additional information about their work.

© 2025 Niu Z et al. *JAMA Network Open*.

eTable 1. Offspring Blood Pressure by Demographics and Maternal Cardiometabolic Risk Factors During Pregnancy

Maternal cardiometabolic risk factor	N	SBP percentile Mean (SD)	DBP percentile Mean (SD)
Any risk factors			
None	6,943	56.68 (26.43)	68.70 (20.83)
Any	5,537	61.39 (26.04)	70.50 (20.46)
p-value		<0.001	<0.001
Gestational diabetes			
No	10,226	57.89 (26.48)	69.43 (20.74)
Yes	805	61.46 (26.16)	70.96 (20.20)
p-value		<0.001	0.11
Hypertensive disorders of pregnancy			
No	10,421	58.32 (26.41)	69.32 (20.69)
Yes	1,693	61.68 (26.34)	71.58 (20.57)
p-value		<0.001	<0.001
Pre-pregnancy obesity			
No	8,584	57.25 (26.28)	68.70 (20.84)
Yes	3,072	61.27 (26.26)	71.07 (20.27)
p-value		<0.001	<0.001
Risk factor combinations			
None	6,943	56.68 (26.43)	68.70 (20.83)
Pre-pregnancy Obesity	1,871	59.89 (26.47)	70.85 (20.23)
GDM	396	60.36 (25.98)	69.44 (20.66)
HDP	874	59.58 (26.47)	70.27 (20.98)
Pre-pregnancy Obesity+ HDP	525	64.06 (26.00)	72.74 (20.59)
Pre-pregnancy Obesity+ GDM	219	62.98 (25.65)	71.95 (19.03)
HDP + GDM	74	63.42 (27.69)	74.33 (20.29)
All three	116	61.06 (26.83)	72.15 (20.46)
p-value		<0.001	<0.001
Maternal race and ethnicity			
Non-Hispanic White	6,522	57.42 (26.08)	67.61 (20.74)
Non-Hispanic Black	1,908	55.10 (27.72)	70.96 (20.98)
Non-Hispanic Asian	856	64.30 (24.65)	74.03 (19.54)
Hispanic	2,305	62.58 (25.93)	71.28 (20.26)
Other	712	60.64 (26.45)	71.60 (20.23)
P value		<0.001	<0.001
Maternal education			
High school or lower	2425	61.61 (26.89)	70.98 (21.12)
Some college, no degree	2971	60.61 (26.33)	70.05 (20.61)
College degree	3781	57.13 (26.20)	68.68 (20.59)
Master degree or higher	2486	57.69 (25.80)	69.26 (20.23)

© 2025 Niu Z et al. *JAMA Network Open*.

P value		0.005	0.001
Family annual income			
<$30,000	1,702	58.57 (27.74)	71.29 (20.21)
$30,000-$49,999	536	61.32 (25.53)	70.60 (20.18)
$50,000-$74,999	525	58.41 (28.25)	70.60 (20.84)
$75,000-$99,999	307	62.49 (24.93)	71.97 (20.05)
$100,000 or more	1,183	63.42 (23.27)	70.97 (20.38)
Don't know	448	60.28 (26.95)	69.19 (19.51)
P value		<0.001	<0.001
Marital status			
Married	6,599	57.98 (26.40)	69.19 (20.65)
Others	1,695	57.10 (28.20)	71.18 (20.36)
Unknown/missing	4,186	60.69 (25.41)	69.31 (20.84)
P value		<0.001	0.002
Parity			
Nulliparous	2,782	59.57 (26.44)	70.31 (20.60)
Parous	8,395	58.27 (26.11)	69.71 (20.56)
P value		0.008	<0.001
Child sex			
Male	6,397	58.26 (26.52)	70.70 (20.54)
Female	6,083	59.31 (26.19)	68.23 (20.76)
P value		0.026	<0.001
Child race and ethnicity			
Non-Hispanic White	5,921	56.92 (26.14)	67.23 (20.75)
Non-Hispanic Black	1,826	54.26 (27.87)	70.67 (21.02)
Non-Hispanic Asian	573	64.61 (24.20)	74.45 (19.39)
Hispanic	2,747	62.64 (25.91)	71.33 (20.30)
Other	1,184	61.84 (25.45)	71.48 (20.13)
P value		<0.001	<0.001
Age category at first blood pressure measure			
2-5 years	8,815	58.91 (26.41)	72.61 (19.53)
6-10 years	2,291	60.40 (24.52)	61.88 (20.66)
11-18 years	1,374	55.10 (28.61)	62.38 (22.72)
P value		<0.001	<0.001

© 2025 Niu Z et al. *JAMA Network Open*.

eTable 2. Sex Modification on the Association Between Maternal Cardiometabolic Risk Factors During Pregnancy and Offspring Blood Pressure

Maternal cardiometabolic risk factor	Male, N=6397	Female, N=6372	P-value for interaction
	Beta (95% CI) on SBP		
Any risk factors	4.99 (3.53 to 6.44)	5.07 (3.68 to 6.46)	0.98
Combinations versus non-risk factors			0.67
Pre-pregnancy obesity only	3.07 (1.16 to 4.98)	3.75 (1.82 to 5.68)	0.68
HDP only	1.28 (-2.34 to 4.91)	3.93 (-0.19 to 8.05)	0.97
GDM only	3.61 (1.03 to 6.20)	3.62 (0.99 to 6.25)	0.26
Pre-pregnancy obesity + HDP	6.57 (3.21 to 9.93)	8.56 (5.34 to 11.78)	0.41
Pre-pregnancy obesity + GDM	6.37 (1.57 to 11.17)	4.95 (0.40 to 10.29)	0.91
HDP + GDM	7.02 (-0.92 to 14.97)	5.96 (-3.12 to 15.04)	0.91
All three	7.42 (0.51 to 14.33)	0.82 (-6.00 to 7.64)	0.21
GDM versus non-GDM	2.84 (0.23 to 5.45)	2.39 (-0.66 to 5.44)	0.31
HDP versus non-HDP	3.77 (1.83 to 5.70)	3.79 (1.87 to 5.72)	1.00
Pre-pregnancy obesity versus non- Pre-pregnancy obesity	3.76 (2.20 to 5.32)	4.48 (2.91 to 6.06)	0.16
	Beta (95% CI) on DBP		
Any risk factors	1.03 (-0.08 to 2.13)	2.73 (1.64 to 3.82)	0.03
Combinations versus non-risk factors			0.27
Pre-pregnancy obesity only	1.59 (0.10 to 3.09)	1.82 (0.26 to 3.37)	0.84
HDP only	-1.06 (-3.90 to 1.77)	0.99 (-2.19 to 4.17)	0.30
GDM only	1.02 (-1.01 to 3.04)	2.72 (0.62 to 4.82)	0.29
Pre-pregnancy obesity + HDP	3.15 (0.53 to 5.78)	4.15 (1.57 to 6.72)	0.56
Pre-pregnancy obesity + GDM	0.72 (-3.05 to 4.49)	3.58 (-0.63 to 7.78)	0.17
HDP + GDM	3.04 (-3.18 to 9.27)	7.29 (0.03 to 14.55)	0.32
All three	2.79 (-2.62 to 8.20)	3.16 (-2.23 to 8.54)	0.83
GDM versus non-GDM	-0.20 (-2.23 to 1.83)	1.77 (-0.52 to 4.06)	0.02
HDP versus non-HDP	1.77 (0.28 to 3.26)	2.83 (1.31 to 4.35)	0.34
Pre-pregnancy obesity versus non- Pre-pregnancy obesity	1.67 (0.46 to 2.88)	2.31 (1.05 to 3.57)	0.46

Model adjusted for maternal age, race/ethnicity, education, income, marital status, parity, and smoking, all during pregnancy.

© 2025 Niu Z et al. *JAMA Network Open*.

eTable 3. Race and Ethnicity Modification on the Association Between Maternal Cardiometabolic Risk Factors During Pregnancy and Offspring Blood Pressure

Maternal cardiometabolic risk factor	Non-Hispanic White, N=6499	Non-Hispanic Black, N=1879	Non-Hispanic Asian, N=855	Hispanic, N=2305	P-value for interaction
			Beta (95% CI) on SBP		
Any risk factors	5.45 (4.02 to 6.87)	5.28 (2.78 to 7.78)	3.31 (-0.45 to 7.07)	4.30 (2.14 to 6.46)	0.58
Combinations versus non-risk factors					0.02
Pre-pregnancy obesity only	4.74 (2.65 to 6.83)	1.64 (-1.49 to 4.76)	6.82 (0.07 to 13.57)	3.20 (0.45 to 5.96)	
HDP only	3.95 (1.57 to 6.33)	5.19 (0.37 to 10.02)	-1.19 (-8.89 to 6.51)	3.74 (-1.31 to 8.80)	
GDM only	0.74 (-3.35 to 4.84)	3.11 (-6.44 to 12.67)	5.00 (-1.15 to 11.16)	0.25 (-6.22 to 6.72)	
Pre-pregnancy obesity + HDP	6.74 (3.42 to 10.06)	12.29 (6.78 to 17.80)	-1.02 (-11.37 to 9.32)	3.59 (-1.52 to 8.70)	
Pre-pregnancy obesity + GDM	3.90 (-1.55 to 9.35)	9.45 (0.50 to 18.40)	5.88 (-4.59 to 16.35)	6.20 (-0.99 to 13.40)	
HDP + GDM	0.55 (-8.24 to 9.34)	19.88 (5.66 to 34.10)	4.89 (-11.45 to 21.24)	11.28 (-4.11 to 26.68)	
All three	4.20 (-2.58 to 10.98)	13.40 (0.22 to 26.59)	-2.22 (-17.88 to 13.43)	2.64 (-9.20 to 14.47)	
GDM versus non-GDM	0.63 (-2.35 to 3.62)	7.60 (2.07 to 13.14)	4.15 (-1.15 to 9.45)	2.19 (-2.34 to 6.73)	0.01
HDP versus non-HDP	3.42 (1.59 to 5.25)	7.48 (4.05 to 10.91)	-2.51 (-8.08 to 3.07)	2.63 (-0.69 to 5.96)	0.01
Obesity versus non-obesity	4.83 (3.19 to 6.47)	3.66 (0.95 to 6.38)	2.88 (-2.03 to 7.79)	3.03 (0.65 to 5.40)	0.03
			Beta (95% CI) on DBP		
Any risk factors	1.33 (0.18 to 2.49)	2.12 (0.17 to 4.06)	3.89 (0.70 to 7.07)	2.78 (1.09 to 4.47)	0.31
Combinations versus non- risk factors					0.07
Pre-pregnancy obesity only	2.48 (0.79 to 4.16)	0.02 (-2.48 to 2.53)	3.02 (-2.31 to 8.34)	1.73 (-0.43 to 3.88)	
HDP only	1.50 (-0.43 to 3.43)	3.70 (-0.23 to 7.63)	6.83 (0.81 to 12.85)	0.17 (-3.78 to 4.12)	
GDM only	-0.69 (-3.98 to 2.60)	0.45 (-7.11 to 8.02)	2.79 (-2.23 to 7.82)	-1.21 (-6.19 to 3.76)	
Pre-pregnancy obesity + HDP	1.81 (-0.90 to 4.52)	6.95 (2.67 to 11.24)	4.82 (-3.36 to 13.00)	5.58 (1.59 to 9.57)	
Pre-pregnancy obesity + GDM	2.69 (-1.76 to 7.14)	1.65 (-5.53 to 8.84)	3.64 (-5.18 to 12.46)	3.75 (-1.79 to 9.29)	
HDP + GDM	3.51 (-3.62 to 10.63)	11.53 (0.38 to 22.68)	11.27 (-1.88 to 24.41)	4.08 (-7.94 to 16.11)	
All three	0.52 (-4.93 to 5.97)	10.06 (-0.19 to 20.31)	2.18 (-10.33 to 14.70)	0.56 (-8.66 to 9.79)	
GDM versus non-GDM	0.14 (-2.21 to 2.48)	3.15 (-1.15 to 7.45)	2.64 (-1.84 to 7.11)	0.36 (-3.07 to 3.79)	0.01

© 2025 Niu Z et al. *JAMA Network Open*.

HDP versus non-HDP	1.37 (-0.11 to 2.84)	5.25 (2.49 to 8.02)	5.35 (0.97 to 9.74)	2.30 (-0.29 to 4.89)	0.07
Obesity versus non-obesity	2.11 (0.82 to 3.41)	0.77 (-1.40 to 2.93)	2.34 (-1.58 to 6.26)	2.80 (0.95 to 4.65)	<0.01

Model adjusted for maternal age, education, income, marital status, parity, and smoking, all during pregnancy.

© 2025 Niu Z et al. *JAMA Network Open*.

eTable 4. Population Characteristics According to Exclusion and Inclusion in Longitudinal Analyses

Variable	Excluded, N = 6,465	Included, N = 6,015	p-value
Maternal demographics			
Maternal age at pregnancy	29.49 (6.79%)	30.42 (5.93%)	<0.001
Maternal race/ethnicity			<0.001
Non-Hispanic White	3346 (52.6)	3176 (53.5%)	
Non-Hispanic Black	1127 (17.7%)	781 (13.2%)	
Non-Hispanic Asian	313 (4.9%)	543 (9.1%)	
Hispanic	1202 (18.9%)	1103 (18.6%)	
Other	377 (5.9%)	335 (5.6%)	
Missing	100	77	
Maternal education			<0.001
High school or lower	1333 (21%)	1092 (18%)	
Some college, no degree	1590 (25%)	1381 (23%)	
College degree	1790 (28%)	1991 (33%)	
Master degree or higher	1282 (20%)	1204 (20%)	
Missing	470	347	
Family annual income			<0.001
<$30,000	1094 (43.7%)	608 (27.6%)	
$30,000-$49,999	287 (11.5%)	249 (11.3%)	
$50,000-$74,999	250 (10.0%)	275 (12.5%)	
$75,000-$99,999	131 (5.2%)	176 (8.0%)	
$100,000 or more	471 (18.8%)	712 (32.4%)	
Don't know	268 (10.7%)	180 (8.2%)	
Missing	3,964	3,815	
Marital status			<0.001
Married	2977 (76.6%)	3622 (82.2%)	
Others	911 (23.4%)	784 (17.8%)	
Unknown/missing	2,577	1,609	
Parity			<0.001
Nulliparous	1332 (23.0%)	1450 (26.9%)	
Parous	4455 (77.0%)	3940 (73.1%)	
Missing	678	625	
Smoking during pregnancy			<0.001
No	5452 (90.6%)	5365 (91.8%)	
Yes	566 (9.4%)	478 (8.2%)	
Missing	447	172	
Maternal cardiometabolic risk factors during pregnancy			
Pre-pregnancy obesity			<0.001
No	4183 (72.7%)	4401 (74.6%)	
Yes	1571 (27.3%)	1501 (25.4%)	
Missing	711	113	
Gestational diabetes			<0.001
No	4826 (92.5%)	5400 (92.9%)	
Yes	392 (7.5%)	413 (7.1%)	
Missing	1,247	202	
Hypertensive disorders of pregnancy			<0.001
No	5282 (86.5%)	5139 (85.6%)	
Yes	826 (13.5%)	867 (14.4%)	

© 2025 Niu Z et al. *JAMA Network Open*.

Missing	357	9	
Offspring characteristics			
Child sex			0.059
Male	3,367 (52%)	3,030 (50%)	
Female	3,098 (48%)	2,985 (50%)	
Child race and ethnicity			<0.001
Non-Hispanic White	3101 (48.0)	2820 (46.9)	
Non-Hispanic Black	1085 (16.8)	741 (12.3)	
Non-Hispanic Asian	218 (3.4)	355 (5.9)	
Hispanic	1395 (21.6)	1352 (22.5)	
Other	568 (8.8)	616 (10.2)	
Missing	98	131	
Age category at first blood pressure measure			<0.001
2-5 years	3,384 (52%)	5,431 (90%)	
6-10 years	1,823 (28%)	468 (7.8%)	
11-18 years	1,258 (19%)	116 (1.9%)	
Blood pressure			
SBP (mmHg)	102.59 (13.06)	95.45 (10.74)	<0.001
DBP (mmHg)	62.91 (9.41)	58.21 (8.69)	<0.001
SBP percentile	60.61 (26.29)	56.79 (26.29)	<0.001
DBP percentile	68.19 (20.70)	70.91 (20.57)	<0.001

Note: * Maternal risk factors here refer to pre-pregnancy obesity, gestational diabetes, and hypertensive disorders of pregnancy; SBP, systolic blood pressure; DBP, diastolic blood pressure.

© 2025 Niu Z et al. *JAMA Network Open*.

eTable 5. Sensitivity Analysis Results Compared With to the Original Results

**Maternal cardiometabolic risk factor**	**SBP**	
	Original	Adjust cohort in GEE model
Any factor	4.71 (3.78, 5.63)	3.21 (2.26, 4.16)
Combinations versus "None"		
Obesity only	3.21 (1.87, 4.55)	2.48 (1.19, 3.77)
HDP only	2.90 (1.06, 4.75)	3.31 (1.54, 5.07)
GDM only	3.68 (1.03, 6.34)	3.25 (0.73, 5.77)
Obesity + HDP	7.38 (5.05, 9.71)	5.89 (3.68, 8.10)
Obesity + GDM	6.30 (2.77, 9.83)	5.17 (1.84, 8.50)
HDP + GDM	6.74 (0.73, 12.75)	6.57 (0.91, 12.23)
All three	4.38 (-0.44, 9.19)	1.90 (-2.66, 6.45)
	**DBP**	
	Original	Adjust cohort in GEE model
Any factor	1.79 (1.06, 2.52)	1.25 (0.48, 2.02)
Combinations versus "None"		
Obesity only	2.15 (1.09, 3.20)	0.83 (-0.21, 1.87)
HDP only	1.57 (0.11, 3.02)	2.23 (0.81, 3.65)
GDM only	0.74 (-1.35, 2.83)	0.84 (-1.19, 2.87)
Obesity + HDP	4.04 (2.20, 5.88)	3.52 (1.73, 5.30)
Obesity + GDM	3.25 (0.45, 6.04)	2.84 (0.14, 5.53)
HDP + GDM	5.62 (0.89, 10.35)	5.44 (0.89, 10.00)
All three	3.45 (-0.34, 7.24)	1.95 (-1.71, 5.62)

Note: *Obesity refers to pre-pregnancy obesity; adjusted model included maternal age, race/ethnicity, education, income, marital status, parity, and smoking, all during pregnancy; HDP, hypertensive disorders of pregnancy; GDM, gestational diabetes; SBP, systolic blood pressure; DBP, diastolic blood pressure.

© 2025 Niu Z et al. *JAMA Network Open*.

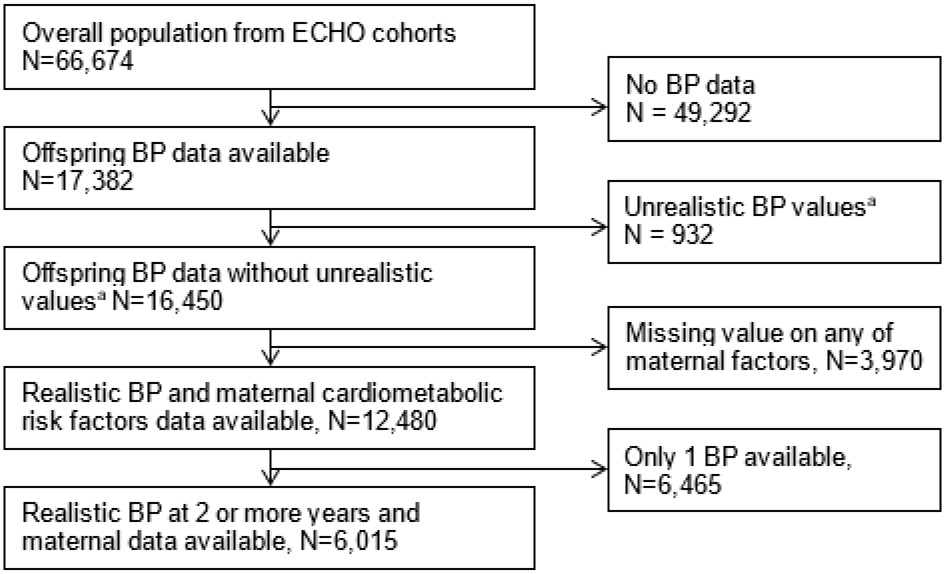

eFigure 1. Flow Diagram of Population Included in the Analysis

[a]: BP values that are out of the range of mean ± 4SD are considered as biologically implausible and more likely a reflection of errors in measurement or data entry; BP, blood pressure.

© 2025 Niu Z et al. *JAMA Network Open*.

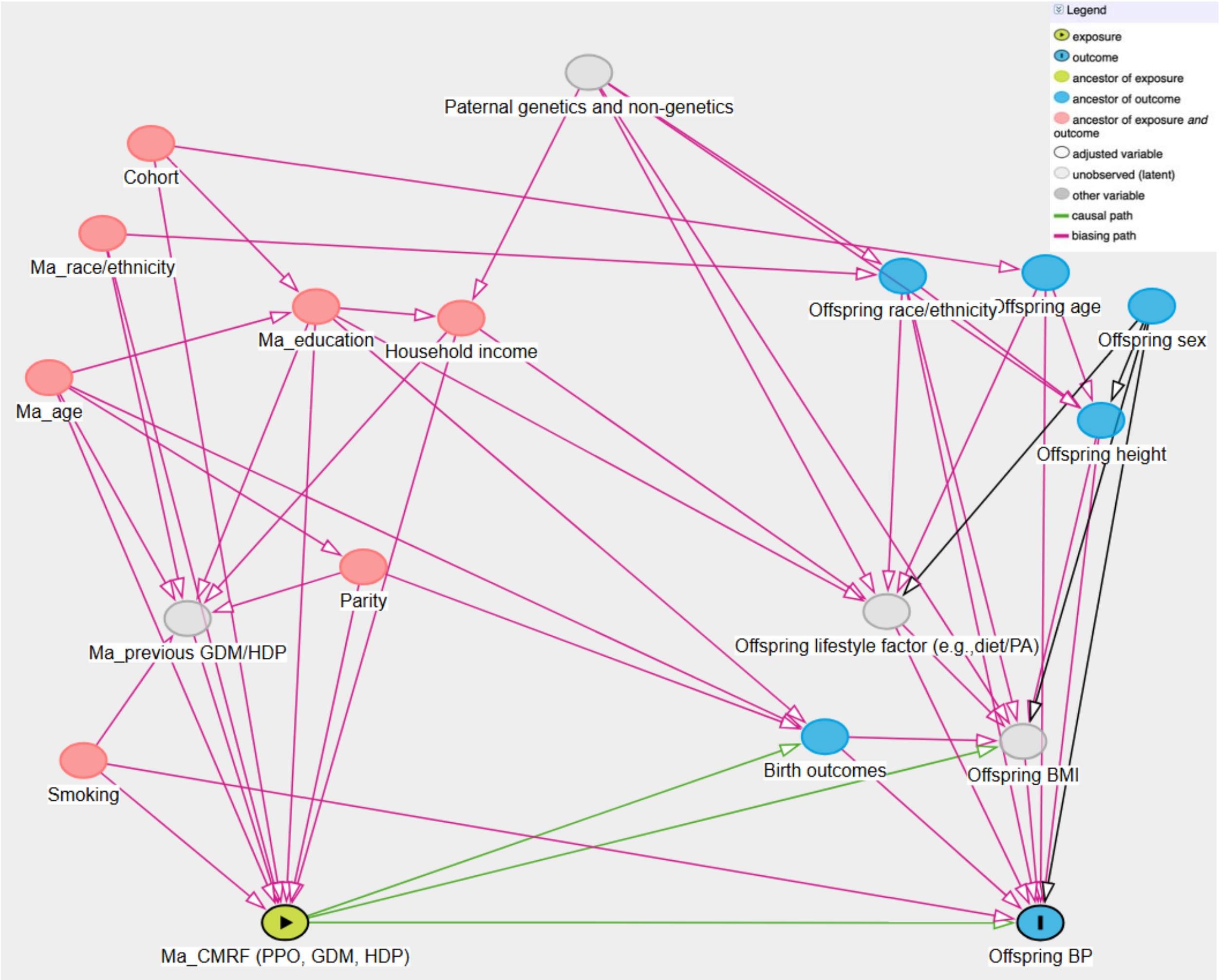

eFigure 2. Causal Diagram

Abbreviations: Ma – maternal, GDM – gestational diabetes, HDP – hypertensive disorder of pregnancy, PPO – pre-pregnancy obesity, CMRF – cardiometabolic risk factors, BMI – body mass index, PA – physical activity, BP – systolic/diastolic blood pressure.

© 2025 Niu Z et al. *JAMA Network Open*.

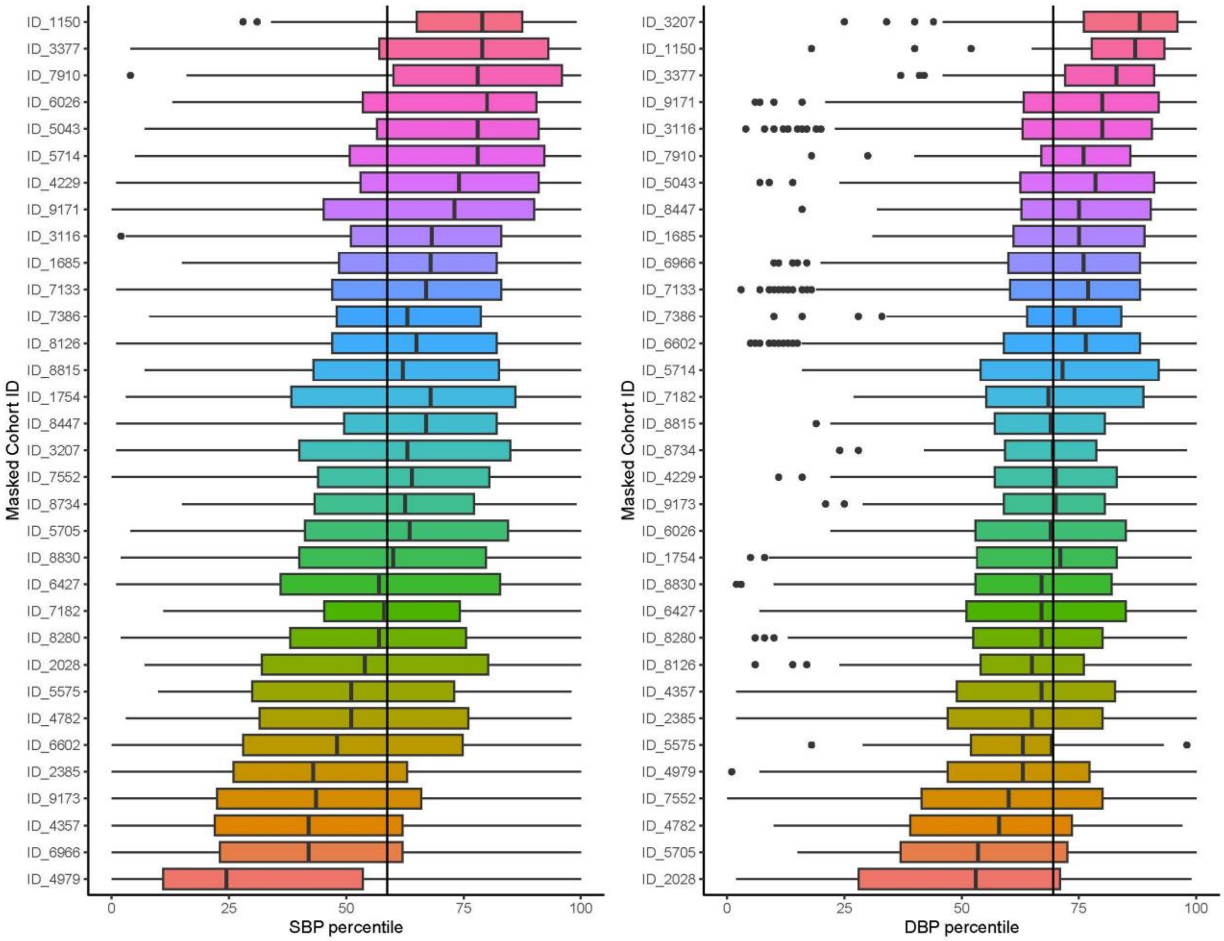

eFigure 3. Blood Pressure Distribution by Cohort
Each box plot represents the spread of BP percentiles (i.e., median, quartiles, and any extreme values) for a single cohort, and the black vertical line indicates the overall mean for the entire analytic sample. Cohort IDs were masked.

© 2025 Niu Z et al. *JAMA Network Open*.

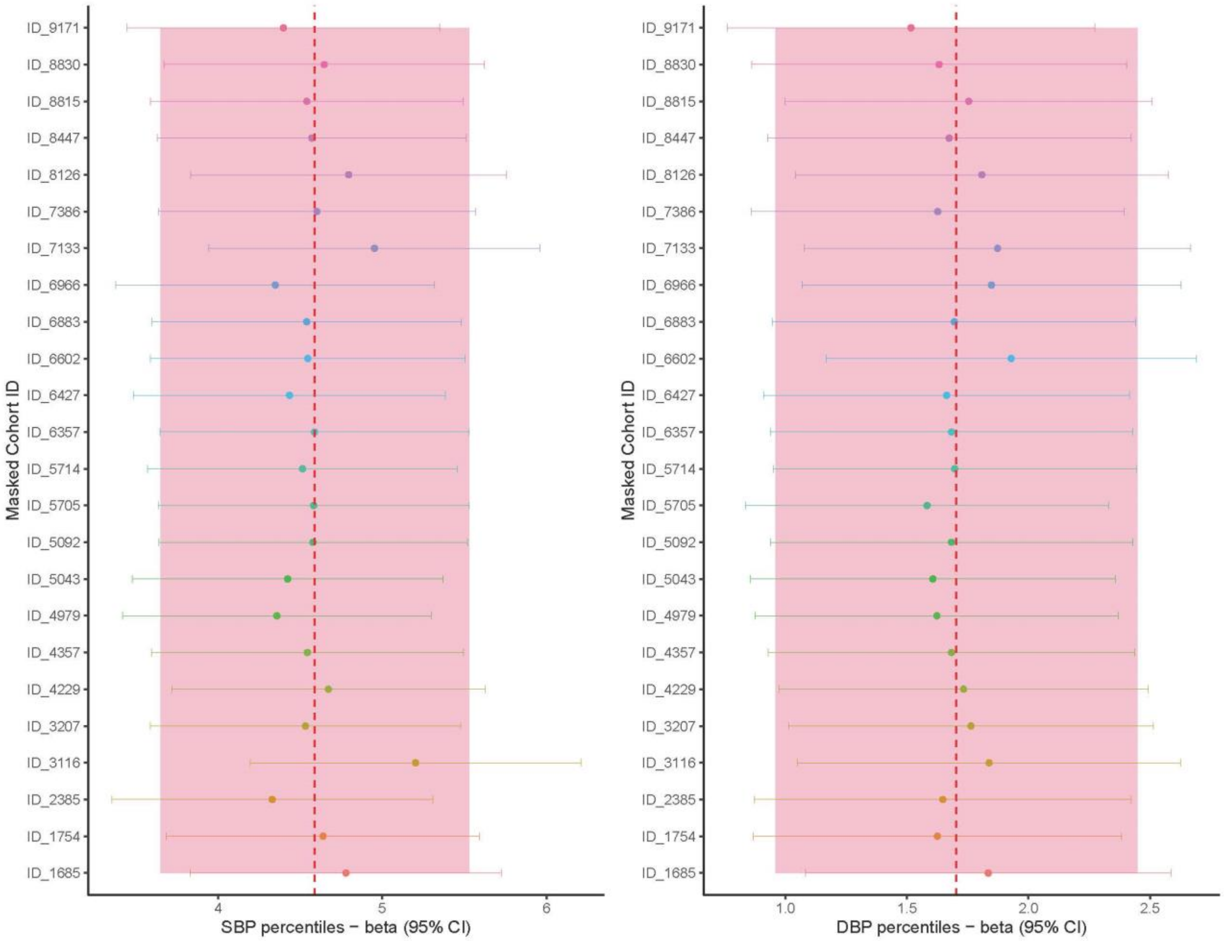

eFigure 4. Beta and 95% CI From Leave-One-Out Analysis

A, Beta and 95% CI for any maternal cardiometabolic risk factors (versus none) from leave one out analysis.
Dashed red line and red shade indicate beta and 95% CI from the overall sample. Model adjusted for maternal age, race/ethnicity, education, income, marital status, parity, and smoking, all during pregnancy. Cohort IDs were masked.

© 2025 Niu Z et al. *JAMA Network Open*.

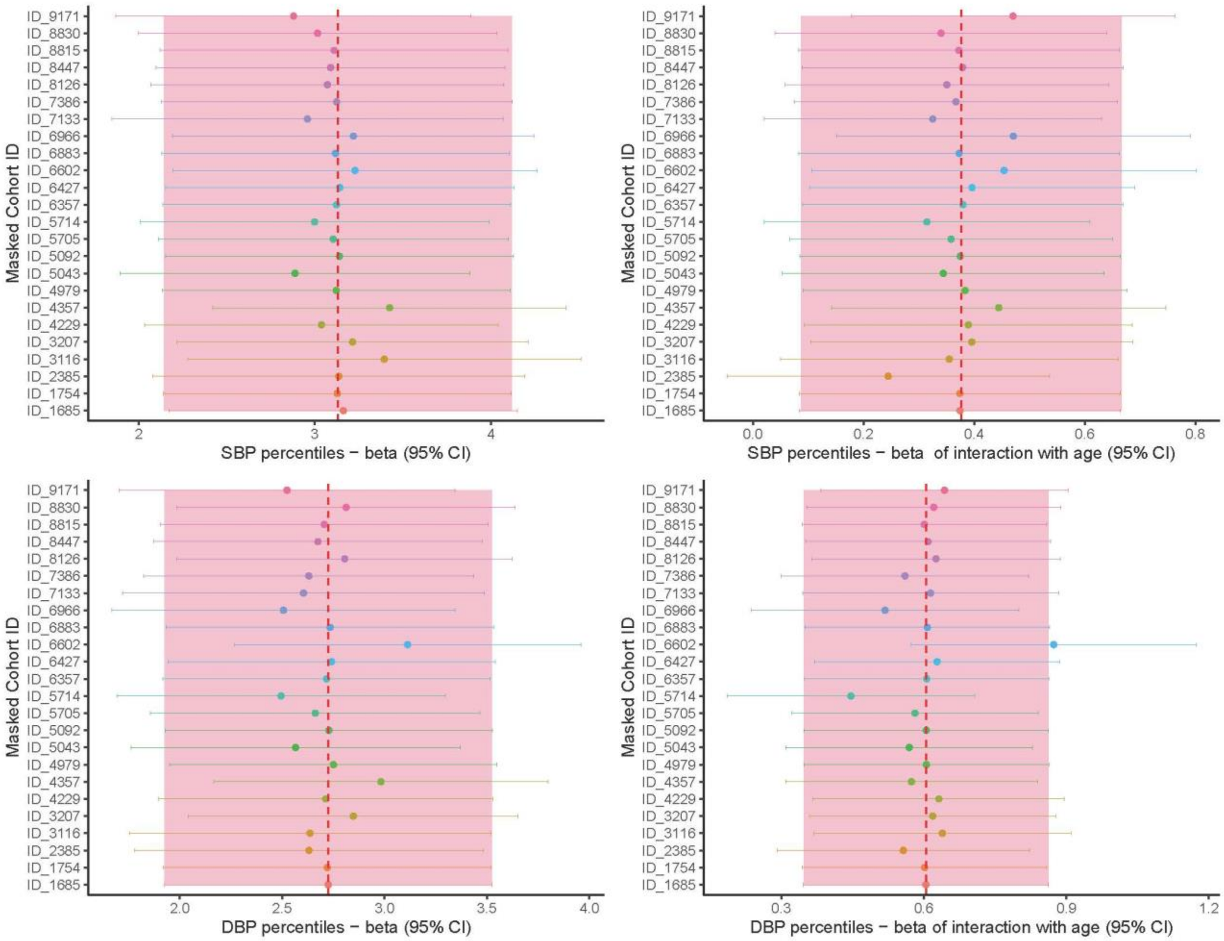

B, Beta (95% CI) of any maternal cardiometabolic risk factors (versus none) and interaction term between any maternal cardiometabolic risk factors and age from leave one out analysis.

Dashed red line and red shade indicate beta and 95% CI from the overall sample. Models adjusted for maternal age, race/ethnicity, education, income, marital status, parity, and smoking, all during pregnancy. Cohort IDs were masked.

© 2025 Niu Z et al. *JAMA Network Open*.
