# Supplementary material for: Maternal Cardiometabolic Risk Factors in Pregnancy and Offspring Blood Pressure at Age 2 to 18 Years
Source: JAMA Netw Open. 2025 May 8;8(5):e259205. doi: 10.1001/jamanetworkopen.2025.9205 (PMC12062903; doi:10.1001/jamanetworkopen.2025.9205)
Supplement: Supplement 3. — Data Sharing Statement [file jamanetwopen-e259205-s003.pdf]

Data Sharing Statement

Niu. Maternal Cardiometabolic Risk Factors in Pregnancy and Offspring Blood Pressure at Age 2 to 18 Years. *JAMA Netw Open*. Published May 08, 2025.
doi:10.1001/jamanetworkopen.2025.9205

Data
Data available: Yes
Data types: Deidentified participant data
How to access data: Data will be publicly available via the ECHO data repository.
When available: With publication

Supporting Documents
Document types: None

Additional Information
Who can access the data: Researchers whose proposed use of the data has been approved.
Types of analyses: For any purpose
Mechanisms of data availability: After approval of a proposal
